# Climate change and health: *atipasunchu allín kausayta*? Can we have health and well-being?

**DOI:** 10.17843/rpmesp.2023.401.12333

**Published:** 2023-03-31

**Authors:** César Cabezas

**Affiliations:** 1 Revista Peruana de Medicina Experimental y Salud Pública, Instituto Nacional, Lima, Peru. Revista Peruana de Medicina Experimental y Salud Pública Instituto Nacional de Salud Lima Peru

Climate change refers to long-term changes in the Earth’s weather patterns, including changes in temperature, rainfall and extreme weather events. Human activities, mainly the burning of fossil fuels - which include carbon dioxide (CO_2_), methane, nitrous oxides and other fluorinated gases - are thought to have caused the concentration of gases leading up to the greenhouse effect. These heat-trapping gases tend to raise global temperatures and increase thermal pollution, which are the main drivers of climate change. In addition, deforestation, urbanization and effluents released by mining, agriculture and manufacturing pollute air, soil and water contributing to climate change. Deforestation, in particular, reduces the land’s ability to remove CO_2_^1^.

In this context, climate change is a major threat to global health, impacting directly and indirectly on society. Heat waves, droughts, severe storms and sea level rise are examples of direct impacts. Heat waves increase mortality rates, as seen in previous El Niño events, by causing hyperthermia associated with sudden temperature changes in young children and older adults. These events can also aggravate cardiovascular and respiratory diseases, as well as to cause injuries due to flooding and activation of streams, as has been occurring in Peru due to the Yaku cyclone, and which will probably happen again with the El Niño phenomenon in the coming months, according to SENAMHI forecasts ^2^.

Indirect impacts include respiratory tract diseases and those related to water and food. For example, a major outbreak of diarrhea by rotavirus was reported in northern Peru this year. On the other hand, ocean warming increases the probability of a reappearance of *Vibrio cholerae* infection, which needs to be diagnosed and treated promptly, otherwise it can have high mortality rates. Dengue fever has been a constant health problem in Peru since 1990. It has spread throughout the country following the distribution of *Aedes aegypti*, which includes the whole Peruvian coastal region, from Tumbes to Tacna, up to the Amazon. The presence of this vector is associated with the accumulation of water in the households or around them, due to rainfall (northern coast and Amazonia), as well as the collapse of drinking water services that forces people to accumulate water inside their homes, producing unintentional breeding sites for mosquitoes (rest of the coast). Insecticides are used every year, both against the larval and adult phases of the mosquitoes; however, resistance to insecticides is an emerging issue. Leptospirosis, a zoonosis that occurs mainly in areas affected by landslides and floods, is frequent due to the exposure of the population to contaminated water. Other indirect problems caused by climate change include food insecurity and malnutrition, forced migration of entire populations, as well as serious damage to housing, labor issues and mental health problems ^3^. All of these events occur currently, as can be evidenced by the sustained increase in the average annual temperature, which affects particularly the most vulnerable populations. This situation is being reported by several outlets, ranging from epidemiological reports to social networks.

In this context, the health sector has an important role to play in setting an example on the road to reducing greenhouse gas emissions. We must invest in healthcare in order to make health facilities more environmentally friendly, through the use of solar panels, energy-efficient equipment and proper waste management. Healthcare infrastructure should be adequate and safe in order to ensure medical attention during and after emergencies and natural disasters ^3^.

The persistence of global warming and the subsequent climate change will produce inevitable and/or irreversible changes, not only to our geography but to our society as a whole if we do not act now. Adaptation measures that are feasible and effective today will become increasingly limited and less useful as time goes by. CO_2_ emissions have to be reduced to zero in order to limit global warming caused by humans. The time to act is now. Deep, rapid and sustained mitigation and accelerated implementation of adaptation measures in this decade would reduce projected losses and damages to humans and ecosystems in the future ^4^. Prioritization of equity, climate justice, social justice, and social inclusion can significantly facilitate mitigation and alleviation of the effects of climate change.

Finally, it is highly relevant to examine our history to learn how our ancestors faced climatic challenges. The Incas were a civilization that adapted to the changing climatic conditions of the Andes for many years, being able to adapt to climatic challenges through the implementation of innovative policies and technologies for their time, such as the construction of agricultural terraces. These terraces allowed farmers to cultivate on steep slopes and avoid soil erosion, but also allowed the control of irrigation and rainwater harvesting, as well as having different altitudes and temperatures for their crops. Another adaptation technique used by the Incas was crop diversification, using different altitudes, climates and ecological niches for different types of food. In addition, the Incas stored provisions in granaries and silos to ensure that they had enough supplies during periods of drought or flood. They also built a network of roads and bridges connecting different parts of the empire, which allowed for the distribution of food and resources throughout their territory, helping them to respond quickly to natural disasters with timely assistance.

**Figure 1:**
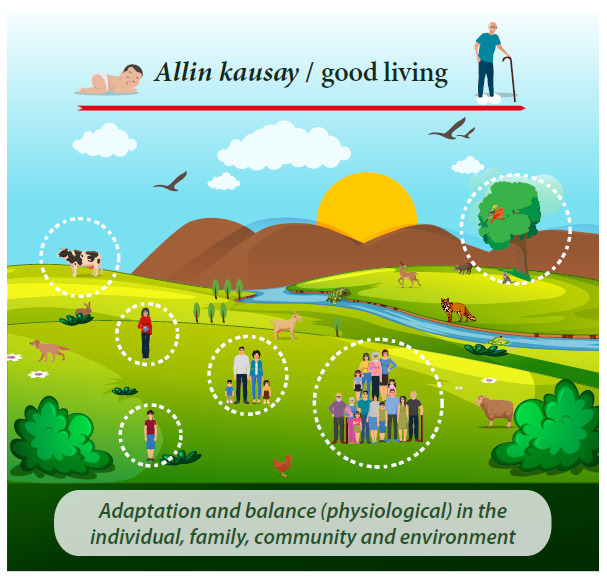
*Allin Kausay* (good living).

Another important ancestral contribution is the conception about life in harmony with nature and the person’s integral balance, including the balance with oneself, with the family, with the community and with the environment ^5^, which was called *Allin kausay* (Figure 1). The definition of *Allin kausay* is aligned with the One Health concept, which constitutes an integrated and unifying approach that aims to balance and optimize in a sustainable manner the health of people, animals and ecosystems ^6^; it is also a strategy to face climate change and its consequences, since it emphasizes prioritizing the health of humans and animals as well as their interaction with the environment. Additionally, *Allin kausay* is aligned with the Sustainable Development Goals (SDGs) ^7^, which are more relevant than ever, and constitute one of the few commitments agreed upon by several countries of the world; nonetheless, many countries are still far from meeting the goals by 2030, which may lead to urgently rethink the initial goals in order to address the current problems.

It is necessary to emphasize that *Allin kausay* was far beyond the mere concept: it was mainly considered a life strategy and was practiced by the citizens of the Inca empire. That is to say, it was not only an objective, but also the way to achieve it. So, will we, their descendants, in this difficult context of climate change, be able to aspire to have the health and wellbeing they strived for? (*Atipasunchu allín kausayta*?). The answer is that now more than ever is the time to address that challenge, before it is too late; and we are running out of opportunities. This requires a global effort to reduce greenhouse gas emissions and promote sustainable practices in industry, agriculture and everyday life, including transitioning to renewable energy sources, improving energy efficiency, adopting sustainable agricultural practices and reducing resource consumption. In addition, we need to embrace One Health perspectives and implement the SDGs. But assuming our individual and collective responsibilities and committing to spreading the message to ensure that the above actions are implemented is just as important. Only then will we have a chance to make the longed-for health and wellness not just a dream, but a reality.
