# Supplementary material for: Climate change and health: atipasunchu allín kausayta? Can we have health and well-being?
Source: Rev Peru Med Exp Salud Publica. 2023 Mar 31;40(1):4–6. doi: 10.17843/rpmesp.2023.401.12333 (PMC10959513; doi:10.17843/rpmesp.2023.401.12333)
Supplement: Translation

# Translation: Cambio climático y salud: *atipasunchu allín kausayta*? ¿podemos tener salud y bienestar?

**Authors:** César Cabezas

**Affiliations:** 1 Revista Peruana de Medicina Experimental y Salud Pública, Instituto Nacional, Lima, Perú.

El cambio climático se refiere a los cambios a largo plazo en los patrones climáticos de la Tierra, incluyendo los cambios en la temperatura, los patrones de precipitación y los eventos climáticos extremos. Se considera que las actividades humanas, principalmente a través de la quema de combustibles fósi*les -*que incluyen dióxido de carbono (CO_2_), metano, óxidos nitrosos y otros gases fluorados- ha provocado el efecto invernadero por concentraciones de gases. Estos gases, al atrapar el calor, tienden a elevar las temperaturas globales y aumentar la contaminación térmica, siendo los principales impulsores del cambio climático. Además, la deforestación, la urbanización y los efluentes liberados por la minería, agricultura y manufactura contaminan el aire, el suelo y el agua y contribuyen al cambio climático. La deforestación, en particular, reduce la capacidad de la tierra para eliminar el CO_2_^1^.

En este contexto, el cambio climático es una gran amenaza para la salud mundial, pues genera impactos directos e indirectos. Entre los primeros podemos enumerar a las olas de calor, sequías, tormentas marcadas e incremento del nivel del mar. Las olas de calor producen un exceso de mortalidad, como se vio en los fenómenos de El Niño previos, produciendo hipertermia asociada a los cambios bruscos de temperatura en niños pequeños y adultos mayores. También pueden producir agravamiento de enfermedades cardiovasculares y respiratorias, así como injurias debidas a inundaciones y activación de quebradas, como viene ocurriendo en el Perú por el fenómeno del Yaku y probablemente se repitan con el fenómeno de El Niño en los próximos meses, según los pronósticos del SENAMHI^2^.

Entre los impactos indirectos, hay que destacar las enfermedades de vías respiratorias y las vinculadas al agua y alimentos. Durante este año, por ejemplo, se ha producido un brote importante de diarrea por rotavirus en el norte del país. Por otro lado, con el calentamiento del agua del mar hay mayor posibilidad de la reaparición del *Vibrio cholerae,* que de no diagnosticarse y manejarse oportunamente, puede producir alta mortalidad entre los afectados. Otro problema de salud casi permanente desde 1990 en el país es el dengue que se viene extendiendo en el territorio nacional de acuerdo a la dispersión del *Aedes aegypti*, que actualmente está prácticamente en toda la costa: desde Tumbes hasta Tacna y en toda la Amazonia. Típicamente, la presencia de este vector está asociada a la acumulación de agua en las casas o en el peridomicilio, tanto por la presencia de lluvias (costa norte y Amazonia), como por el colapso de los servicios de agua potable que obliga a los pobladores a acumular agua dentro de las viviendas, produciendo criaderos involuntarios para los mosquitos (resto de la costa). Todos los años se utilizan insecticidas, tanto para la fase larvaria como para la fase adulta del mosquito, sin embargo, la complicación que tenemos actualmente es la aparición de resistencia a insecticidas. Por otro lado, la exposición de la población a aguas contaminadas también hacen frecuente la presencia de leptospirosis, una zoonosis presente en todas las zonas afectadas por los huaycos y desbordes. Otros problemas indirectos que se originan a raíz del cambio climático son la inseguridad alimentaria y la desnutrición, las migraciones forzadas de poblaciones enteras, así como serias afectaciones de las viviendas, problemas laborales y compromiso de la salud mental de las personas damnificadas ^3^. Lo más resaltante de todos estos efectos es que vienen ocurriendo actualmente, puesto que ya podemos constatar que el incremento sostenido de la temperatura media anual está afectando a las poblaciones, sobre todo a las más vulnerables, situación que se ve reflejada en fuentes que van desde los reportes de epidemiología hasta las redes sociales.

En este contexto, el sector salud debe desempeñar un papel importante en brindar el ejemplo en el camino hacia la reducción de las emisiones de gases de efecto invernadero. Debemos realizar inversiones para que los establecimientos de salud sean más amigables con la ecología, a través del uso de paneles solares, equipos que optimicen la eficiencia energética y una adecuada gestión de residuos. Además debemos garantizar la seguridad de la infraestructura sanitaria que la atención sea continua durante y luego de las emergencias y los desastres naturales ^3^. 

La persistencia del calentamiento global y el consecuente cambio climático producirá cambios inevitables y/o irreversibles, no solo en nuestra geografía sino en nuestra sociedad en conjunto si no actuamos desde ahora. Las opciones de adaptación que hoy nos resultan factibles y efectivas, se volverán cada vez más limitadas y menos útiles. Limitar el calentamiento global causado por nosotros, los humanos, requiere reducir las emisiones netas de CO_2_ a cero. Es momento de actuar: la mitigación profunda, rápida y sostenida y la implementación acelerada de acciones de adaptación en esta década reducirían las pérdidas y daños proyectados para los humanos y los ecosistemas en el futuro ^4^. La priorización de la equidad, justicia climática, justicia social, y la inclusión social pueden facilitar de manera significativa la mitigación y alivio de los efectos del cambio climático.

Finalmente, resulta sumamente relevante examinar nuestra historia para conocer cómo nuestros ancestros enfrentaron los desafíos climáticos. Los incas fueron una civilización que se adaptó a las condiciones climáticas cambiantes de los Andes durante muchos años, siendo capaces de adecuarse a los retos climáticos a través de la implementación de políticas y tecnologías innovadoras para su época, como la construcción de andenes agrícolas. Estos andenes permitían a los agricultores cultivar en laderas empinadas y evitar la erosión del suelo, pero también permitían el control del riego y la recolección del agua de lluvia, así como tener diferentes altitudes y temperaturas para sus sembríos. Otra técnica de adaptación a la que recurrieron los incas fue la diversificación de cultivos, utilizando diferentes alturas, climas y nichos ecológicos para diferentes tipos de alimentos. Además, los incas almacenaban provisiones en graneros y silos para asegurarse de tener suficientes suministros durante los períodos de sequía o inundación. También construyeron una red de caminos y puentes que conectaba diferentes partes del imperio, lo que permitía la distribución de alimentos y recursos en todo su territorio, ayudándolos a responder rápidamente a los desastres naturales con ayuda oportuna.

Otro aporte ancestral que debiéramos tener en cuenta es la concepción que ellos tenían sobre la vida en armonía con la naturaleza y el equilibrio integral de la persona, incluyendo el equilibrio consigo mismo, con la familia, con la comunidad y con el medio ambiente ^5^, al que se denominó *Allin kausay* (Figura 1). Su definición, está alineada al concepto sanitario de Una Salud o *One Health*, que constituye una aproximación integrada y unificadora que tiene como meta balancear y optimizar de manera sostenible la salud de las personas, animales y ecosistemas ^6^^)^ y que en la práctica es también una estrategia para enfrentar al cambio climático y sus secuelas, dado que pone énfasis en priorizar la salud de los humanos y los animales y su interacción con el medio ambiente. Adicionalmente, el *Allin kausay* está alineado a los objetivos de desarrollo sostenible (ODS) ^7^, que siguen vigentes más que nunca, y constituyen uno de los pocos compromisos consensuados por los diferentes países del mundo, pero que todavía muchos países están lejos de cumplir para el 2030, debiendo replantear las metas inicialmente trazadas, pero con la urgencia que se necesita para afrontar los problemas vigentes.

**Figura 1:** Allin Kausay (buen vivir).

Es preciso enfatizar que el *Allin kausay* iba mucho más allá del mero concepto: era sobre todo tomado como una práctica por los ciudadanos del imperio incaico y una estrategia de vida en sí. Es decir, no solo era un objetivo, sino al mismo tiempo era el camino para cumplirlo. Entonces, al final ¿podremos nosotros, sus descendientes, en este difícil contexto del cambio climático aspirar a tener la salud y bienestar por la que ellos se esforzaban? (*Atipasunchu allín kausayta*?). La respuesta es que hoy más que nunca es el momento de abordar ese desafío, antes que sea demasiado tarde. Se nos agotan las oportunidades. Para ello, se requiere por un lado, de un esfuerzo global en reducir las emisiones de gases de efecto invernadero y promover prácticas sostenibles en la industria, la agricultura y la vida cotidiana, incluyendo la transición a fuentes de energía renovable, la mejora de la eficiencia energética, la adopción de prácticas agrícolas sostenibles y reducción del consumo de recursos. Además, tenemos que adoptar las perspectivas de *One Health* e implementar los ODS. Pero tan importante como ello también es asumir nuestra responsabilidad individual y colectiva para contribuir al cambio que necesitamos y comprometernos con difundir el mensaje de velar para que se implementen las acciones arriba mencionadas. Solo así tendremos una oportunidad para que la salud y el bienestar tan anhelados no sólo sean un sueño, si no una realidad.
